# Household cockroaches carry CTX-M-15-, OXA-48- and NDM-1-producing enterobacteria, and share beta-lactam resistance determinants with humans

**DOI:** 10.1186/s12866-019-1629-x

**Published:** 2019-12-04

**Authors:** Noah Obeng-Nkrumah, Appiah-Korang Labi, Harriet Blankson, Georgina Awuah-Mensah, Daniel Oduro-Mensah, Judelove Anum, James Teye, Solomon Dzidzornu Kwashie, Evariste Bako, Patrick Ferdinand Ayeh-Kumi, Richard Asmah

**Affiliations:** 1 Department of Medical Laboratory Sciences, School of Biomedical and Allied Health Sciences, P.O. Box KB 143, Accra, Ghana West Africa; 2 Department of Microbiology, Korle-Bu Teaching Hospital, P.O. Box 77, Accra, Ghana West Africa; 3 Molecular and Experimental Mycobacteriology, Research Centre Bostel, Leibniz Lung Center, Parkallee 1-40, 23845 Borstel, Germany; 4 School of Life Sciences, University of Nottingham, England, NG7 2UH UK; 5 Department of Applied Chemistry and Biochemistry, University for Development Studies, Navrongo Campus, P.O. Box 24, Navrongo, Ghana West Africa; 6 Department of Biochemistry and Microbiology, University of Ouagadougou, Ouagadougou, Burkina Faso

**Keywords:** Cockroach, ESBL, Carbapenemase, Multilocus sequence typing, Conjugation

## Abstract

**Background:**

This study was designed to investigate whether household cockroaches harbor cephalosporin-resistant enterobacteria that share resistance determinants with human inhabitants. From February through July 2016, whole cockroach homogenates and human fecal samples from 100 households were cultured for cephalosporin-resistant enterobacteria (CRe). The CRe were examined for plasmid-mediated AmpC, ESBL, and carbapenemase genes; antibiotic susceptibility patterns; and conjugative transfer of antibiotic resistance mechanisms. Clonal associations between CRe were determined by multi-locus sequence typing (MLST).

**Results:**

Twenty CRe were recovered from whole cockroach homogenates from 15 households. The prevalence of households with cockroaches that harbored CRe, AmpC- (based on phenotype, with no identifiable *bla*AmpC genes), ESBL-, and carbapenemase-producers were 15, 4, 5%(2 *bla*_CTX-M-15/TEM-1_; 1 *bla*_CTX-M-15/TEM-4_; 1 *bla*_TEM-24_; 1 *bla*_SHV-4_) and 3%(2 *bla*_NDM-1_ genes and 1 *bla*_OXA-48_ gene), respectively. Overall, 20 CRe were recovered from 61 fecal samples of inhabitants from all 15 households that had cockroach samples positive for CRe. Of these, 5CRe (1 per household) were positive for ESBLs (*bla*_TEM-24_, *bla*_TEM-14_, *bla*_CTX-M-15/TEM-4_, *bla*_SHV-3_, *bla*_CTX-M-15/TEM-1_) and none carried AmpCs or carbapenemases. From 4% of households, the pair of cockroach and human CRe shared the same sequence type (ST), clonal complex (CC), antibiogram, and conjugable *bla* gene sequence (house 34, *E. coli* ST9/CC20-*bla*_TEM-4_; house 37, *E. coli* ST44/CC10-*bla*_CTX-15/TEM-4_; house 41, *E. coli* ST443/CC205-*bla*_CTX-15/TEM-1_; house 49, *K. pneumoniae* ST231/CC131-*bla*_SHV-13_).

**Conclusion:**

The findings provide evidence that household cockroaches may carry CTX-M-15-, OXA-48- and NDM-1-producers, and share clonal relationship and beta-lactam resistance determinants with humans.

## Background

Production of extended-spectrum beta-lactamases (ESBL), Class C cephalosporinases and carbapenemases constitutes the primary antibiotic resistance mechanism in Enterobacteriaceae [1, 2]. Together, these beta-lactamases confer resistance to all available beta-lactam antibiotics, and are associated with significantly high levels of co-resistance to other classes of broad spectrum antimicrobials [1–6]. In several regions in Africa, the CTX-M-15 type ESBLs are becoming increasingly predominant [7, 8]. Occurrence of the recently described OXA-48-type carbapenemase, and widespread reports of *bla*_NDM-1_ genes across Africa, further compound the outlook of the antibiotic resistance problem [9–13]. Indeed, emergence and spread of such resistance determinants in bacteria is often related to abuse of beta-lactam antibiotics [14, 15]. However, distribution and persistence of the resistant pathogens may be hugely aided by poor sanitation [16–18]. Cockroaches, which widely colonize the environment, including human dwellings, may well act as vehicles for antibiotic-resistant bacteria.

Cockroaches are common in many households and are known to harbor an array of pathogens, some of which may be carriers of drug-resistance determinants including beta-lactamases [19–23].They often reside in household sewage pipe systems, which are a repository of diverse infectious microorganisms. However, only few studies have investigated AmpC, ESBL or carbapenemase resistance elements in bacteria from cockroaches [24]. Consequently, the vector potential of cockroaches in the dissemination of beta-lactamase-related drug resistance mechanisms is largely under-reported. This study was designed to determine whether cephalosporin resistant enterobacteria recovered from cockroaches in a household were identical to those that colonized human inhabitants from the same household □ with particular focus on clonal relationships, beta-lactam-resistance mechanisms, conjugability of resistance determinants and antibiogram.

## Results

**Fig. 1:**
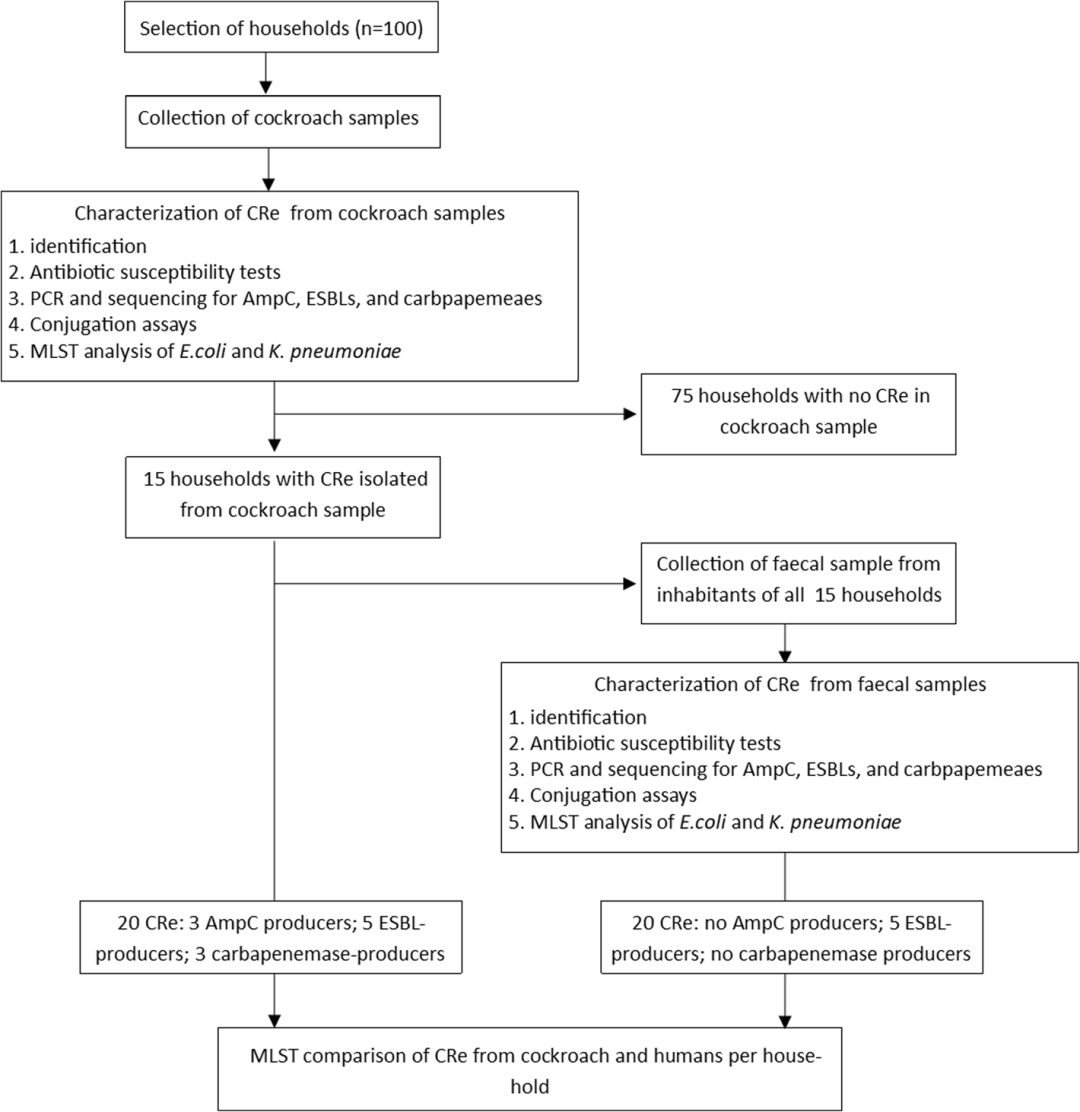
Summary of study protocols and outcomes

A flowchart of the study outcomes in shown in Fig. 1. All 100 insect homogenates yielded polymicrobial cultures after inoculation on SSI agar with 30 μg cefpodoxime disks. Cockroach samples from 15 households had homogenate culturesscreen positive for CRe. Ten of the samples grew one dominating CRe colony type and 5 had two morphologicaly distinct dominant CRe colonies with different antibiogram □ corresponding to 20 screen positive Enterobacteriaceae isolates (Table 1). The 20 isolates were each resistant to cefotaxime or ceftazidime, and were assigned a CRe phenotype. From the inhabitants of the 15 households that had cockroach samples positive for CRe, a total of 61 fecal samples were collected. The average number of inhabitants per household was 4 ± 1.3. Twenty (32.8%) of the 61 faecal samples were screen positive for CRe on SSI agar plate. The 20 fecal cultures each yielded only one dominant CRe colony type □ corresponding to 20 screen positive CRe (1 isolate per faecal sample) with different antibiogram. All 20 screen-positive CRe were resistant to cefotaxime or ceftazidime, and were also assigned a CRe phenotype. Ten and 5 households respectively had 1 and 2 inhabitants fecally colonized by CRe (Table 2).

**Table 1:**
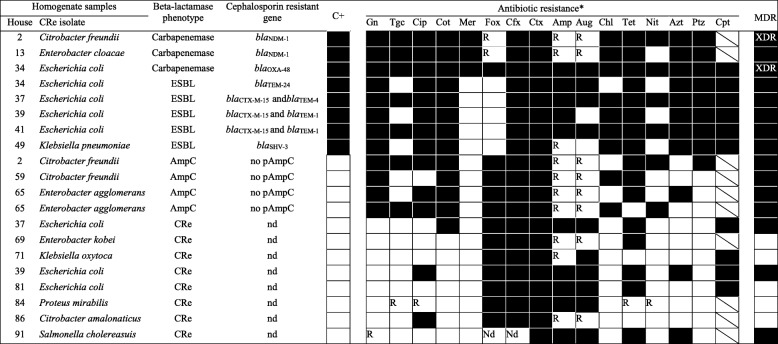
Antibiogram of CRe recovered from whole insect homogenates

*As per column headings, dark cells indicate “yes”; white cells indicate “no” C+, successfully transferred the beta-lactamase encoding genotype to J53 *E.coli* recipient via conjugation;CRerefers to cephalosporin resistant Enterobacteriaceae with no detectable phenotype for AmpC, ESBL or carbapenemases; Gn, gentamicin; Tgc, tigecycline; Cip, ciprofloxacin; Cot, cotrimoxazole; Mer, meropem; Fox, cefoxitin; Cfx, cefuroxime; Ctx, cefotaxime; Amp, ampicillin; Aug, aumentin; Chl, chloramphenicol; Tet, tetracycline; Nit, nitrofurantoin; Azt, aztreonam; Aug, amoxicillin/clavulanic acid, Ptz, piperacillin/tazobactam; Cpt, ceftaroline (approved for only *E.coli*, *K. pneumoniae*, *K* oxytoca. For cells with diagonal lines, Cpt is not approved for testing organism); pAmpC, plasmid mediated AmpC gene; MDR, multidrug resistanct; XDR, extensively drug resistant; R, not reported (test organisms intrinsically resistant to antibiotic); nd, no detectable plasmid AmpC, ESBL or carbapenemase gene

**Table 2:**
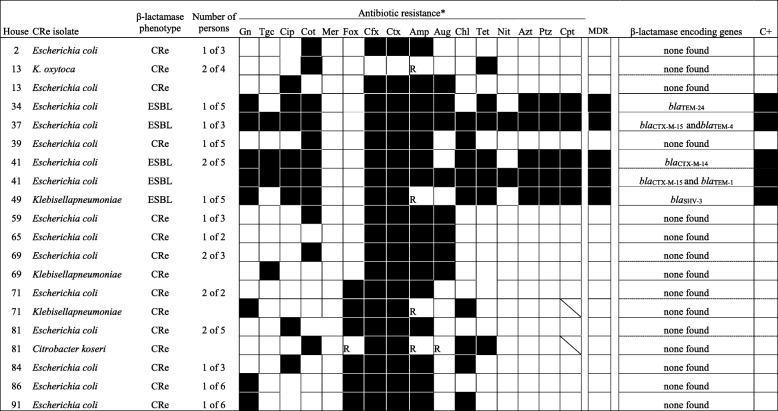
Antibiogram of CRe recovered from human inhabitants in households with cockroach CRe

*As per column headings, dark cells indicate “yes”; white cells indicate “no” C+, successfully transferred the beta-lactamase encoding genotype to J53 *E.coli* recipient via conjugation; CRe refers to cephalosporin resistant Enterobacteriaceae with no detectable phenotype for AmpC, ESBL or carbapenemases; Gn, gentamicin; Tgc, tigecycline; Cip, ciprofloxacin; Cot, cotrimoxazole; Mer, meropem; Fox, cefoxitin; Cfx, cefuroxime; Ctx, cefotaxime; Amp, ampicillin; Aug, aumentin; Chl, chloramphenicol; Tet, tetracycline; Nit, nitrofurantoin; Azt, aztreonam; Aug, amoxicillin/clavulanic acid, Ptz, piperacillin/tazobactam; Cpt, ceftaroline (approved for only *E.coli*, *K. pneumoniae*, *K* oxytoca. For cells with diagonal lines, Cpt is not approved for testing organism); pAmpC, plasmid mediated AmpC gene; MDR, multidrug resistanct; XDR, extensively drug resistant; R, not reported (test organisms intrinsically resistant to antibiotic); nd, no detectable plasmid AmpC, ESBL or carbapenemase gene

### Characterization of CRe

#### Cockroach samples

Of the 20 CRe, 12 isolates (from 10 samples) expressed ESBL (*n* = 5), AmpC (*n* = 4) or were resistant to meropenem (*n* = 3) (Table 1). The AmpC-producers were *Enterobacter freundii* (*n* = 2) and *Enterobacter agglomerans* (*n* = 2). Isolates with ESBL phenotype were 4 *E. coli* and 1 *K. pneumoniae*. Two of the 3 meropenem resistant isolates (*E. freundii* and *C. cloacae*) were found to be class B metallo-beta-lactamase (MBL)-producers. The third meropenem resistant isolate (*E. coli*) was non-susceptible to temocillin (30 μg), and was deemed presumptively positive for OXA-48 like carbapenemase (Table 1). None of the CRe isolates was positive for any combination of the three phenotypes. Of the 5 ESBL-producers, PCR and nucleotide sequencing identified *bla*_CTX-M-15/TEM-1_ in 2 *E. coli* isolates (Table 1). The remaining 3 harbored either *bla*_CTX-M-15/TEM-4_, *bla*_TEM-24_ or *bla*_SHV-3_ ESBL genes. None of the AmpC-producers had an identifiable plasmid-mediated AmpC gene. The 2 MBL-positive isolates each carried *bla*_NDM-1_ gene. The meropenem resistant *E. coli* with non-susceptibility to temocillin carried *bla*_OXA-48_ gene. Thus, the overall prevalence of households with cockroaches that harbored CRe, ESBL-, AmpC- and carbapenemase-producers were respectively 15, 5, 4 and 3%. Antibiogram of the 20 CRe revealed differences in resistance patterns between AmpC-, ESBL-, or carbapenemase-positive isolates and those 8 CRe negative for any of the 3 phenotypes (Table 1). Multi drug-resistant (MDR) phenotype was indicated in all isolates with AmpC, ESBL or carbapenemase phenotype. Two of the 3 carbapenemase-positive CRe were XDR. Amongst the 8 CRe negative for the three phenotypes, 3 showed MDR phenotype.

#### Human faecal samples

Of the 20 CRe, none was positive for AmpC or carbapenemase phenotype. Five CRe (1 per household) expressed ESBLs (4 *E. coli* and 1 *K. pneumoniae*). When the ESBL-producing faecal isolates were subjected to PCR and sequencing, the *K. pneumoniae* carried *bla*_SHV-3_. The 4 *E. coli* separately harbored *bla*_TEM-24_, *bla*_TEM-14_, *bla*_CTX-M-15/TEM-4_, and *bla*_CTX-M-15/TEM-1._ The antibiotic susceptibility profile of all 20 human CRe mirrored that observed for cockroach isolates □ with obvious differences in resistance pattern between ESBL- and non-ESBL producers (Table 2).

### Conjugation assays

All CRe from cockroach and human samples were subjected to conjugation experiments. Successful conjugation events were demonstrated only in ESBL- or carbapenemase-producing isolates (Table 3). For these isolates, PCR amplification and sequencing of the ESBL or carbapenemase genes from the *E. coli* J53 transconjugants showed the same *bla* gene types previously identified in the donor isolates. The ESBL genes in human isolates transferred at slower conjugation frequencies (range: 1.1 × 10^− 5^-1.9 × 10^− 4^) compared to those from cockroach isolates (range: 2.3 × 10^− 3^-4.8 × 10^− 2^). For cockroach isolates, carbapenemase genes appeared to conjugate with lower frequencies (range: 1.1 × 10^− 3^-1.9 × 10^− 3^) compared to the ESBL genes (range: 2.3 × 10^− 3^-4.8 × 10^− 2^). Resistance to non-β-lactam antimicrobials was co-transferred in some cases (Table 3). The most frequently co-transferred phenotypes were resistance to cotrimoxazole, tetracycline and ciprofloxacin. Tigecycline and nitrofurantoin resistance did not transfer to recipients despite repeated attempts. All CRe isolates negative for ESBL or carbapenemase could not transfer their cephalosporin resistance to *E. coli*j53 recipients. Similarly, none of the AmpC-producing isolates transferred the phenotype. In both cases, there was no further susceptibility testing to other antibiotics after the CRe phenotype was found to be absent in recipients.

**Table 3:**
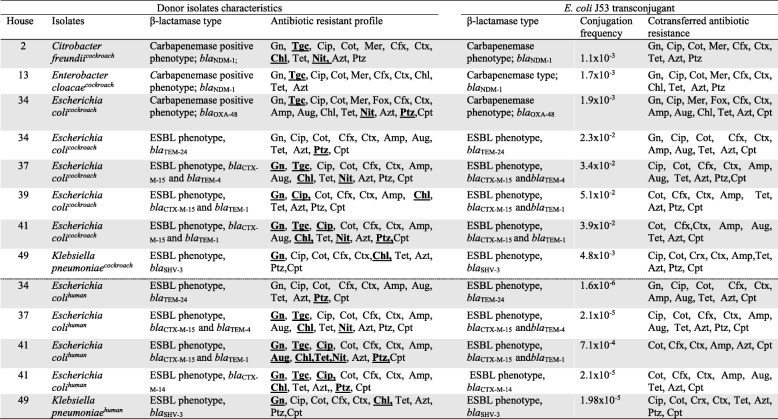
Conjugation characteristics of ESBL- and carbapenemase-producing CRe isolated from cockroaches and humans

^*^Highlighted antibiotics were not cotransferred to transconjugants; Gn, gentamicin; Tgc, tigecycline; Cip, ciprofloxacin; Cot, cotrimoxazole; Mer, meropem; Fox, cefoxitin; Cfx, cefuroxime;Ctx, cefotaxime; Amp, ampicillin; Aug, aumentin; Chl, chloramphenicol; Tet, tetracycline; Nit, nitrofurantoin; Azt, aztreonam; Aug, amoxicillin/clavulanic acid, Ptz, piperacillin/tazobactam, Cpt, ceftaroline (approved for only *E.coli*, *K. pneumoniae*, *K oxytoca*)

### Phylogenetic analysis

**Fig. 2:**
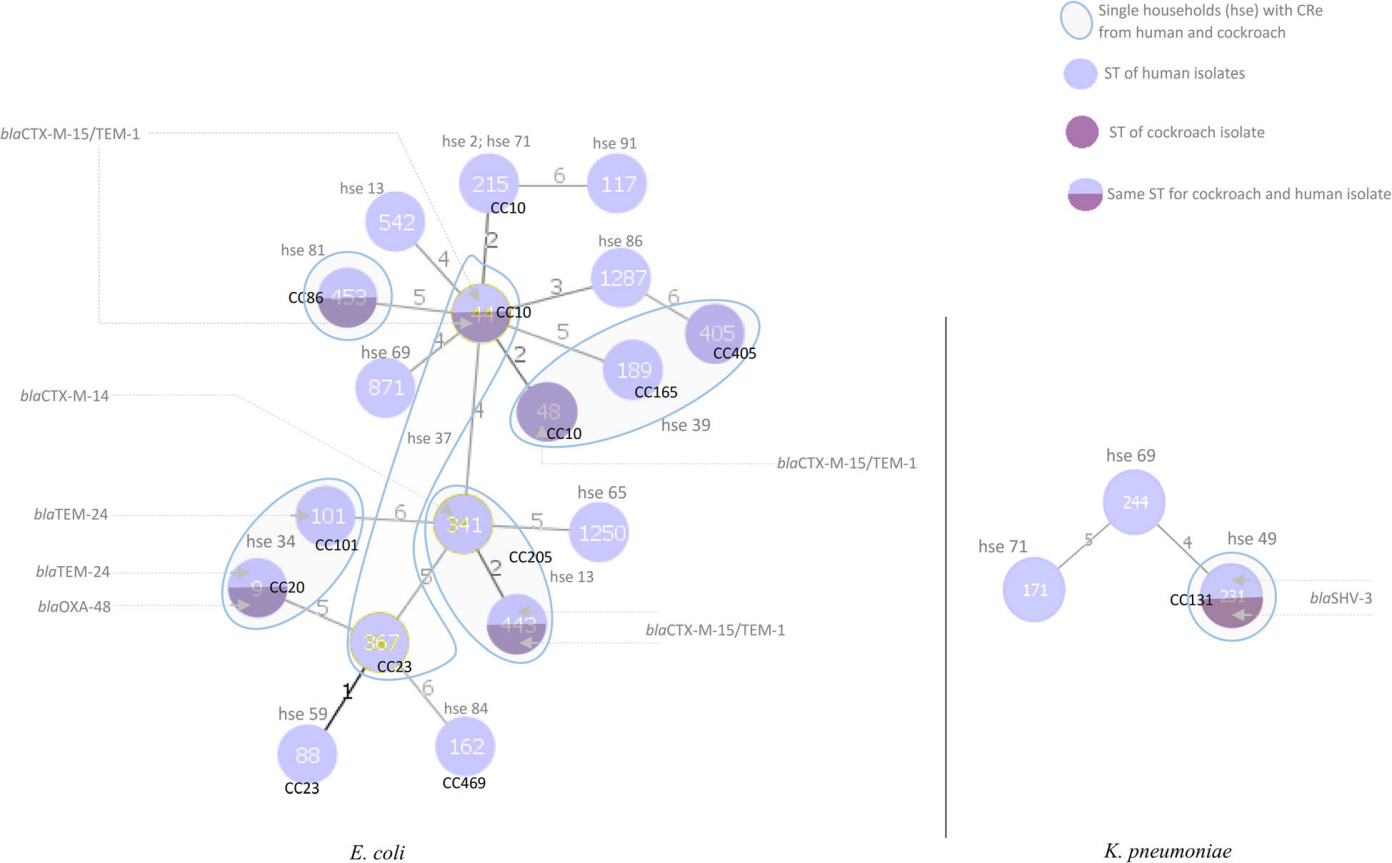
Minimum spanning tree based on MLST allelic profiles of cephalosporin resistant *E. coli* and *K. pneumoniae*from human and cockroach samples. Each circle represents an identified MLST sequence type (ST). The circle in red (lower half) represents an ST identified in the cockroach CRe. The circle in blue (the upper half) represents an ST identified in the human CRe. The numbers on the connecting lines illustrate the number of differing alleles. The clonal complexes (CC) if present are indicated for the STs. The pAmpC, ESBL or carbapenemase genes if present are indicated for the CRe from each household

Our focus was to determine whether CRe cultured from household cockroaches were identical to those which colonized human inhabitants. There were 20 cockroach CRe belonging to 15 households with 20 human CRe. The MLST was considered if CRe from cockroach and human samples per household belonged to the same bacterial species, or were *E. coli* and *K. pneumoniae*. Eighteen human CRe (15 *E. coli* and 3 *K. pneumoniae*) and 9 cockroach CRe (8 *E. coli* and 1 *K. pneumoniae*) from the 15 households were thus included. Of the 15 households, 6 had human and cockroach CRe of the same bacterial species (*E. coli* or *K. pneumoniae*) (Table 4). From 4 of the 6 households, the pair of human and cockroach CRe shared the same ST and *bla*_ESBL_ gene sequence (house 34, *E. coli* ST9-*bla*_TEM-4_; house 37, *E. coli* ST44-*bla*_CTX-15/TEM-4_; house 41, *E. coli* ST443-*bla*_CTX-15/TEM-1_; house 49, *K. pneumoniae* ST231-*bla*_SHV-13_). The pairs also had the same antibiogram; and successfully transferred their ESBL phenotype and genotype by conjugation to *E. coli* J53 recipients (Table 3). In 1 of the 6 households (house 81), the pair of human and cockroach CRe belonged to the same *E. coli* ST453but differed in antibiogram and had no identifiable ESBL, AmpC or carbapenemase genes. The pair did not transfer their cephalosporin resistance phenotypes by conjugation to *E. coli* J53 recipients. In another of the 6 households (house 47), a human inhabitant was colonized by *E. coli* ST341-*bla*_CTX-M-14_ which was different from the corresponding cockroach CRe (*E. coli* ST443 with *bla*_CTX-15/TEM-1_ ESBL gene). Both isolates however belonged to the same clonal complex CC205. Generally, we observed low intra-species similarity regardless of beta-lactamase gene sequences (Fig. 2). Four *E. coli* STs, belonging to different clonal complexes, were found in only cockroach isolates (ST48/CC10, ST101/CC101, ST367/CC23, ST405/CC405). Similarly, 5 *E. coli* singleton STs (ST117, ST542, ST871, ST1250, ST1287) and 5 *E. coli* STs with associated clonal complexes (ST88/CC23, ST162/CC469, ST189/CC165, ST215/CC10, ST341/CC205) and were found in human isolates only. The *E. coli* ST215/CC10 was identified in 2 human isolates from separate households. The following STs with associated clonal complexes were detected in both human and cockroach isolates: *E. coli*; ST9/CC20, ST44/CC10, ST443/CC205, ST453/CC86 and *K.* pneumonia; ST231/CC86). The *K. pneumoniae* STs identified from only human CRe were ST171 and ST244 singletons.

**Table 4:** MLST analysis of CRe from cockroach and human inhabitants per household

Household code		Isolates	MLST	
			ST	CC
2		*E. coli* ^*human*^	215	10
2		*C. freundii*^*cockroach*^	–	
2		*C. freundii* ^*cockroach*^	–	
13		*K. oxytoca* ^*human*^	–	
13		*E. coli* ^*human*^	542	singleton
13		*E. cloacae* ^*cockroach*^	–	
34		*E. coli* ^*human*^	9	20
34		*E. coli* ^*cockroach*^	9	
34		*E. coli* ^*cockroach*^	101	101
37		*E. coli* ^*human*^	44	10
37		*E. coli* ^*cockroach*^	44	
37		*E. coli* ^*cockroach*^	367	23
39		*E. coli* ^*human*^	189	165
39		*E. coli* ^*cockroach*^	48	10
39		*E. coli* ^*cockroach*^	405	405
41		*E. coli* ^*human*^	341	205
41		*E. coli* ^*human*^	443	
41		*E. coli* ^*cockroach*^	443	
49		*K. pneumoniae*^*human*^	231	131
49		*K. pneumoniae* ^*cockroach*^	231	
59		*E. coli* ^*human*^	88	23
59		*C. freundii* ^*cockroach*^	–	
65		*E. coli* ^*human*^	1250	singleton
65		*E. agglomerans*^*cockroach*^	–	
65		*E. agglomerans*^*cockoach.*^	–	
69		*E. coli* ^*human*^	871	singleton
69		*K. pneumoniae* ^*human*^	244	singleton
69		*E. kobei* ^*cockroach*^	–	
71		*E. coli* ^*human*^	215	10
71		*K. pneumoniae* ^*human*^	171	singleton
71		*K. oxytoca* ^*cockroach*^	–	
81		*E. coli* ^*human*^	453	86
81		*C. koseri* ^*human*^	–	
81		*E. coli* ^*cockroach*^	453	86
84		*E. coli* ^*human*^	162	469
84		*P. mirabilis* ^*cockroach*^	–	
86		*E. coli* ^*human*^	1287	*singleton*
86		*C. amalonaticus* ^*cockroach*^	–	
91		*E. coli* ^*human*^	117	singleton
91		*S. cholaeraesuis* ^*cockroach*^	–	

*C+, conjugation tests; dark cells within the C+ column show that the isolate successfully transferred their β-lactamase phenotype and genotype to J53 *E.coli* recipient via conjugation; white cells within the C+ column indicate unsuccessful conjugation of β-lactamase phenotype and genotype to J53 *E.coli* recipient via conjugation; MLST, multilocus sequence typing; ST, Sequence Type; CC, clonal complex

## Discussion

This study investigated whether within individual households, cockroaches and humans may share bacterial isolates of the same clone, and also antibiotic resistance determinants of public health concern. Generally, interpretation of this data is driven by findings from other reports that have indicated direct transfer of ESBL-producing bacteria to humans through close contact with animal sources [25–27]. A number of reports implicate insects, including cockroaches, in the carriage and spread of antibiotic resistant bacteria [28–32]. This is the first published description of blaNDM-1 and blaOXA-48 carbapenemases in enterobacteria recovered from household cockroaches.

Four observations from this study merit special attention. First, evidence is presented to demonstrate that household cockroaches may carry drug-resistant isolates, including multidrug-resistant *bla*_CTX-M-15_-, extensively drug-resistant *bla*_OXA-48_- and extensively drug-resistant *bla*_NDM-1_-producing enterobacteria. In human dwellings, cockroaches may move about freely but are usually found breeding in bathrooms, toilets and cupboards for storing food [28, 33]. Outside the home, they are commonly observed in drains and around damaged septic tanks. Their preferred living quarters, coupled with the fact that they are omnivorous, brings them in frequent contact with stored foods as well as sewage and different kinds of biological wastes [28, 34]. The isolation of these bacteria in household cockroaches is alarming and of public health concern, given that the strains could very quickly disseminate and initiate a pandemic spread of clones for which effective antibiotics may not be readily available. The ease of transmission of *bla*_NDM-1_ genes has already been described [2, 35–37]. Interestingly, although the identified *bla*_NDM-1_ and *bla*_OXA-48_ genes were conjugable, no household inhabitant was fecally colonized with a carbapenem resistant isolate. In Ghana, there are no published data on carbapenemases, but it is the experience that carbapenem resistance is low [6, 38, 39].

Second, about 4% of households had cockroach and human CRe that shared the same sequence type and clonal complex, antibiogram, *bla*_ESBL_ gene sequence, and successfully transferred their ESBL phenotypes and genotypes by conjugation. These findings suggest insect-mediated transmission by clonal spread. The presence of cephalosporin-resistant *Salmonella choleraesuis* in household cockroach, which is a pig typhoid strain, is a strong indication of the extent to which cockroaches could carry infectious contamination from diverse sources to humans. Cockroaches are known to be a common pest of confined pigs [40, 41] and have been shown to acquire and harbour bacterial pathogens from pigs [42]. Acquisition of bacterial pathogens by insects, from farm or domestic animals, is documented [43–45]. In the study site, as in many neighborhoods in rural and urban Africa, domestic and/or farm animals, including dogs, chicken and goats, may roam freely. The relatively high fecundity of cockroaches, coupled with the fact that mature insects may live for up to 2 years or more in association with humans [46], highlight household cockroaches as constituting a formidable ‘revolving door’ through which ESBL-producing enterobacteria may disseminate to human contacts.

Third, the successful inter-genus conjugation events reported in this study suggest that, at least for the non-*E. coli* isolates used as donors, the plasmids involved are likely to be conjugative plasmids with broad host range. This hints at the potential for these resistance determinants to spread freely across bacterial species in the environment, including the possibility of rescue of susceptible bacteria during antibacterial stress [47].

Last, we did not detect AmpCs, ESBLs, or carbapenemases in 8 of 12 cockroach CRe and 15 of 20 human CRe with resistance phenotypes to 3rd-generation cephalosporins. The results show a clear separation, in which the level of resistance to various antibiotics was higher among strains expressing ESBL, AmpC or carbapenemase than those negative for the enzymes. Similarly, resistance mechanisms to several other non-beta-lactam antibiotics were cojugated in parralel with ESBL and carbapenemase genotypes. The CRe isolates without ESBL, AmpC or carbapenemase were susceptible to several antibiotics tested. None of these CRe also successfully transferred their cephalosporin resistance phenotype by conjugation. The results may suggest that this CRe cluster of isolates harbour chromosomal-borne 3rd-generation cephalosporin-resistant determinants with limited functional spectrum compared to AmpCs, ESBLs or carbapenemases [1–6]. The findings highlight from the clinical point of view that, if we can succeed at curtailing the spread of ESBL-, AmpC-, or carbapenemase producing enterobacteria, we may somewhat succeed at reducing the prevalence of antimicrobial resistance among enterobacteria.

Our data should be interpreted considering some potential limitations. We accept the possibility that the cockroach isolates reported in this study may be of transient colonization. However, it is notable that the cockroach CRe were the only dominant colonies in culture□ suggesting stable colonization rather than the consequence of sudden external contamination. The data on the complimentary sampling of humans included only inhabitants of households where CRe was cultured from cockroach samples. Due to the small sample size and provincial household concentration, our data is not meant to be representative of the human prevalence of AmpC-, ESBL-, or carbapenemase-producing bacteria in the study site or similar locations in Ghana. Suffice it to say that faecal carriage of beta-lactamase-producing enterobacteria in the Ghanaian community setting is the focus of another manuscript under consideration for publication elsewhere. Nevertheless, it is noteworthy that a profiling of antibiotic resistance patterns in Accra, Ghana indicated high incidence of cephalosporin resistance in antibiotic resistant bacteria [48]. It is the experience in Ghana that resistance to commonly used antibiotics in primary health care is high, and the prevalence is rising. Although antibiotic use is a known risk factor for the emergence of antibiotic resistance, we did not collect data on antibiotic use by study participants for this report. Information on antibiotic therapy would have better helped to delineate the faecal carriage patterns of household inhabitants with the endemic beta-lactam resistance in the community, their interrelated factors for spread, and the correlating role of household cockroaches as important reservoir of resistant genes. Notwithstanding the shortcomings, our findings highlight the importance of cockroaches as a potential reservoir of epidemiologically significant multidrug resistant pathogens of public health concern.

## Conclusion

We report the alarming colonization of household cockroaches with multidrug resistant CTX-.

M-15 ESBL-producers, and extensively drug resistant NDM-1 and OXA-48 carbapenemase-positive *Enterobacteriacaea*. Our findings highlight cockroaches as insects of public health concern, and call for regulations on their control, especially in healthcare settings.

## Methods

### Study design and setting

Between February and July 2016, cockroaches and human fecal samples were collected from 100 households in Ashaley Botwe, an urban municipality in Accra, Ghana. The municipality has a population of approximately 78,215, with most households occupied by an average of 5.6 persons [49]. The major source of water is pipe borne, and most of the households have proper biological waste disposal systems with flush toilets and standardized septic tanks. Bathwater and liquid wastes from kitchens may, however, be observed running freely in open drains. Households included in the study were at least 150 m from each other. Hundred households were selected by systematic random sampling using the Kish method [50] which statistically allowed for equal chances of selecting any household in the community. From each household, live indoor cockroaches were collected. At the same time, all inhabitants per household were requested to self-collect and submit stool samples. The participants provided written, informed consent. The study received ethical approval from the Ethics and Protocol Review Committee of the School of Biomedical and Allied Health Sciences, University of Ghana, with approval identification number: SBAHS-MD./10,512,194/aa/5a/2016–2017.

### Sample collection and processing

Households were provided with cockroach collection kits (sterile 100 mL containers with screw caps, sterile zipper bags, sterile gloves, sterile entomological forceps) and guidelines for collection to avoid any human contamination. A member from each household was trained to capture the cockroaches. Only live cockroaches found indoors were collected for this study. From each household, four cockroaches regardless of species were requested and pooled as one sample. Each cockroach sample was soaked in 40 mL Brain Heart Infusion broth (Sigma, UK), vortexed vigorously for approximately 5 min, and ground with a sterile rod. The triturate was again vortexed vigorously for 6 min to obtain a whole insect homogenate. A loop-full (approximately 10 μL) of suspension from each sample was inoculated onto SSI agar plate (SSI, Diagnostica, Denmark) with 30 μg cefpodoxime disks (MAST, UK) and incubated overnight at 37 °C. For stool samples, 1 g of each specimen was suspended in 10 mL of sterile 0.9% physiological saline and vigorously vortexed. 1 mL of the suspension was cultured on SSI agar plateswith 30 μg cefpodoxime disks as described above. From each culture plate, distinct morphological phenotypes of enterobacteria growing within an inhibition zone of ≤21 mm for cefpodoxime were considered as screen positive for third-generation cephalosporin resistance. Each distinct morphological phenotype was identified to the species level using biochemical test kits Minibact-E® (SSI, Diagnostica, Denmark) according to the manufacturer’s guidelines. Subsequently, four colonies of each species were tested for confirmation of CRe status by Kirby-Bauer disk diffusion using cefotaxime (30 μg) and ceftazidime (30 μg) antibiotic disks, per guidelines of the Clinical and Laboratory Standards Institute (CLSI) [51]. Isolates resistant to cefotaxime or ceftazidime were confirmed as third-generation cephalosporin resistant (CRe).

### Susceptibility test and assays for ESBL, AmpC and carbapenemase

Confirmed CRe isolates were tested for susceptibility to the following antibiotic disks (MAST, UK) according to CLSI guidelines [51]: ampicillin (10 μg), augmentin (10/260 μg); meropenem (10 μg), tetracycline (30 μg), chloramphenicol (30 μg), cotrimoxazole (100/240 μg), gentamicin (10 μg), ciprofloxacin (5 μg), nitrofurantoin (100 μg); piperacillin/tazobactam (10/30 μg), cefoxitin (30 μg), ceftaroline (30 μg), and tigecycline (30 μg).*Klebsiella pneumoniae* ATCC 700603 and *Escherichia coli* ATCC 25922 were used as quality control strains. Ceftaroline breakpoint was interpreted with European Committee for Antibiotic Susceptibility Testing (EUCAST) guidelines [52]. Phenotypic detection of ESBL production was by the combination disk method with cefotaxime (30 μg) or ceftazidime (30 μg), alone or in combination with clavulanic acid (10 μg) [53]. AmpC expression was suspected in isolates with reduced susceptibility (inhibition zone < 20 mm) to cefoxitin (30 μg). AmpC confirmation was by susceptibility testing to cefotaxime (30 μg) or ceftazidime (30 μg) antibiotic disks, with or without boronic acid (250 μg) as per the manufacturer’s guidelines (Rosco Diagnostica, Taastrup, Denmark). Isolates with an increase of ≥5 mm in zone diameter, due to boronic acid, were considered AmpC positive. Isolates with inhibition zone of < 21 mm to meropenem(10 μg) were considered carbapenem resistant [53]. Carbapenem resistant isolates were confirmed for class A and B carbapenemases using boronic acid (600 μg) and EDTA (750 μg) effects, respectively, on meropenem (10 μg). Strains with boronic acid or EDTA effect of ≥5 mm increase in zone diameter were considered positive for class A or B carbapenemase phenotype, respectively. Carbapenem resistant strains that were not susceptible to temocillin (30 μg) were considered positive for class D carbapenemase. Carbapenem resistant strains were also subjected to the Modified Hodges Test [53].

### Conjugation

All CRe isolates were included in the conjugation assay. Conjugations were done using sodium azide-resistant *E. coli* J53 as recipient [54]. None of the CRe isolates showed resistance to sodium azide. The donors were cultured in Luria-Bertani (LB) (MAST, UK) broth with cefotaxime (8 μg/mL) overnight. The recipient was also cultured overnight in LB broth but with no antibiotic. Subsequently, 1 mL aliquots of each donor and the recipient were separately transferred into fresh 10 mL LB broth and incubated for 2 h at 37 °C. For each donor, 100 μL of culture was mixed with an equal volume of the recipient and the mixture was incubated for 6 h at 37 °C. Selection for transconjugants was on MacConkey agar supplemented with sodium azide (150 mg/L) and cefotaxime (8 mg/L) or cefoxitin (32 mg/L) or meropenem (2 mg/L). Transconjugants were confirmed for AmpC, ESBL or carbapenemase phenotype as previously described.

### Genotypic characterization

Gene amplification and sequencing were done for ESBL-, AmpC-, and carbapenemase-producing isolates as well as their transconjugants. For each isolate, 10 μL of pure culture on Mueller Hinton agar was suspended in 300 μL Milli-Q® water, heated for 10 min at 98 °C, and subsequently centrifuged for 5 min at 4 °C and 20,000×g. The supernatant DNA lysate was transferred into sterile 1.5 mL Eppendorf® tubes for storage at − 5 °C. See Additional file 1: Table S1 for amplification primers and conditions. For ESBLs, PCR was performed for *bla*_TEM_, *bla*_SHV_, *bla*_CTX-M-1_, *bla*_CTX-M-2_, *bla*_CTX-M-9_, *bla*_OXA-2_, *bla*_OXA-10_. Isolates with AmpC phenotypes were examined for 6 families of plasmid-mediated AmpC genes including MOX, CMY, DHA, ACC, EBC and FOX using the multiplex assay [55]. For carbapenemases, PCRs were designed for specific genes belonging to class A, B, and D carbapenemase phenotypes. A multiplex PCR assay was performed to differentiate5 genes (GES, KPC, SME, INI-NMC-A) for class A carbapenemase, and 6 genes (IMP, VIM, GIM, SPM, SIM, and NDM-1) for class B phenotypes [56]. Isolates with Class D carbapenemase phenotypes were examined for OXA-48 like genes. Additional internal primers (Additional file 1: Table S1) were used for sequencing CTX-M-1, CTX-M-9, SHV and TEM genes. Nucleotide and deduced protein sequences were compared with sequences in the NCBI database (http://www.ncbi.nlm.nih.gov/BLAST). TEM and SHV beta-lactamase sequences were compared to wild-type *E. coli* TEM-1 and SHV-1 at http://www.lahey.org/studies.

### Phylogenetic analysis

Multilocus sequence typing (MLST) was conducted when cockroach and human CRe per household belonged to the same bacterial species, or were *E. coli* and *K. pneumoniae*. Isolates that satisfied the inclusion criteria were *E. coli* and *Klebsiella pneumoniae*. The previously described *E. coli* protocol [57] and the Institut Pasteur scheme for *K. pneumoniae* (http://bigsdb.pasteur.fr/klebsiella/) were used. Seven housekeeping genes were amplified and sequenced for each *E. coli* (adk, fumC, gyr, icd, mdh, purA and recA) and *K. pneumoniae* (gapA, infB, mdh, pgi, phoE, rpoB, tonB) isolates. Sequences of the seven house-keeping genes were analysed using the CodonCode Aligner software version 8.1 (Germany). The MLST sequence types (ST) and clonal complexes (CC) were assigned in accordance with the online platform PubMLST database (https://pubmlst.org/). Phylogenetic minimum spanning tree was constructed using the online programme PHYLOViZ [58].

### Data analyses

Data were entered into a Microsoft Excel spreadsheet for analysis by proportions and percentages. Multidrug-resistant (MDR) isolates were those resistant to ≥1 agent in ≥3 antimicrobial categories (aminoglycosides, flouoroquinolones, penicillins, penicillins/β-lactamase inhibitors, antipseudomonal penicillins/ β-lactamase inhibitors, cephamycins, anti-MRSA cephalosporins, 1st and 2nd generation cephalosporins, 3rd and 4th generation cephalosporins, monobactams, carbapenems, polymixins, phosphonic acids, folate-pathway inhibitors, glycylcyclines, phenicols). Extensively drug-resistant (XDR) isolates were non-susceptible to ≥1 agent in all but ≤2 antimicrobial categories. Conjugation frequency per recipient was expressed by dividing the number of transconjugants by the initial number of recipients.

## Supplementary information

**Additional file 1: Table S1.** Oligonucleotides and PCR conditions used for amplification (internal primers included for sequencing).

## Data Availability

The dataset for the current study is available from the corresponding author on reasonable request.

## Abbreviations

AmpCs: Class C cephalosporins
ATTC: American Type Culture Cells
CC: Clonal complex
CLSI: Clinical and Laboratory Standards Institute
CRe: Cephalosporin resistant enterobacteria
EDTA: Ethylene diamine tetraacetic acid
ESBLs: Extended-spectrum beta-lactamases
LB broth: Luria-Bertani Broth
MBL: Metallo-beta-lactamases
MLST: Multilocus Sequence Typing
PCR: Polymerase Chain Reactions
SSI: StatensSserum Agar
TC: American Type culture Cells
