# Supplementary material for: Household cockroaches carry CTX-M-15-, OXA-48- and NDM-1-producing enterobacteria, and share beta-lactam resistance determinants with humans
Source: BMC Microbiol. 2019 Dec 4;19:272. doi: 10.1186/s12866-019-1629-x (PMC6894353; doi:10.1186/s12866-019-1629-x)
Supplement: Supplementary file 1 — Additional file 1: Table S1. Oligonucleotides and PCR conditions used for amplification (internal primers included for sequencing). [file 12866_2019_1629_MOESM1_ESM.docx]

# **Supporting information**

S1 Table.Oligonucleotides and PCR conditions used for amplification (internal primers included for sequencing)

| Primer (target) Primer sequences (5’-3’) | | T ^o^C ^a^ at 90^o^C | PCR cycles |
| --- | --- | --- | --- |
| *ESBLs* | |  |  |
| TEM (918bp) | FP: GTATCCGCTCATGAGACAATAACCCTG  RP: CCAATGCTTAATCAGTGAGGCACC  Internal FP CCGGAGCTGAATGAAGCCAT  Internal RPCGTTGTTGCCATTGCTGCAG | 63^o^C | 30 |
| SHV (842bp) | FP: CGC CTG TGT ATT ATC TCC CTG TTAGCC  RP: TTG CCA GTG CTC GAT CAG CG  Internal FP : ACCATGAGCGATAACAGCGC  Internal RP: AAGCGCCTCATTCAGTTCCG | 63^o^C | 30 |
| OXA-2 (330bp) | FP: gttaacaggggctttgcagg  RP: TGCACGCAGTATCCAGTTGC | 63^o^C | 30 |
| OXA-10 (655bp) | FP: ATGAAAACATTTGCCGCATATGTA  RP: ACACCAGGATTTGACTCAGTTCC | 63^o^C | 30 |
| CTX-M-1(940 bp) | FP: GACAGACTATTCATGTTGTTGTTAWTTCG  RP: CCGTTTCCSCTATTACAAA  Internal FP: GGACGATGTCACTGGCTGAG  Internal RP: TTTCGTCTCCCAGCTGTCGGG | 50 ^o^C | 30 |
| CTX-M-2 (253 bp) | FP: acagttggtgacgtggcttaagg  RP: TCAGAAACCGTGGGTTACGA | 50 ^o^C | 30 |
| CTX-M-8/25/26 (690/346bp) | FP1: ACATCGCGTTAAGCGGAT  FP2: GCACGATGACATTCGGG  RP: AACCCACGATGTGGGTAGC | 50 ^o^C | 30 |
| CTX-M-9 (860bp) | FP: atggtgacaaagagagtgcaacg  RP: ATGATTCTCGCCGCTGAAGC  Internal FP: caaattgattgcccagctcg  Internal RP: AAACGTCTCATCGCCGATCG | 50 ^o^C | 30 |
| *AmpCs* | |  |  |
| MOX (520 bp) | FP: GCTGCTCAAGGAGCACAGGAT  RP: CACATTGACATAGGTGTGGTGC | 64^o^C | 25 |
| CIT (462 bp) | FP: TGGCCAGAACTGACAGGCAAA  RP: TTTCTCCTGAACGTGGCTGGC | 64 ^o^C | 25 |
| DHA (405 bp) | FP: AACTTTCACAGGTGTGCTGGGT  RP: CCGTACGCATACTGGCTTTGC | 64 ^o^C | 25 |
| ACC (346 bp) | FP: AACAGCCTCAGCAGCCGGTTA  RP: TTCGCCGCAATCATCCCTAGC | 64 ^o^C | 25 |
| EBC (302 bp) | FP: TCGGTAAAGCCGATGTTGCGG  RP: CTTCCACTGCGGCTGCCAGTT | 64 ^o^C | 25 |
| FOX (190bp) | FP: AACATGGGGTATCAGGGAGATG  RP: CAAAGCGCGTAACCGGATTGG | 64 ^o^C | 25 |
| CMY (1100 bp) | FP: AAATCGTTATGCTGCGCTCT  RP: GACACGGACAGGGTTAGGAT  Internal FP: AAATTAAGCTCAGCGATCCG  Internal RP: CAGCGGGCCATATCAATAAC | 64 ^o^C | 25 |
| *Metallo-beta-lactamases (Class B Carbapenemases)* | |  |  |
| NDM (760bp) | FP: GAAGCTGAGCACCGCATTAG  FP: TGCGGGCCGTATGAGTGATT | 52 ^o^C | 30 |
| VIM (390bp) | FP: GATGGTGTTTGGTCGCATA  RP: CGAATGCGCAGCACCAG | 52 ^o^C | 30 |
| GIM (477bp) | FP: TCGACACACCTTGGTCTGAA  RP: AACTTCCAACTTTGCCATGC | 52 ^o^C | 30 |
| SIM (570bp) | FP: TACAAGGGATTCGGCATCG  RP: TAATGGCCTGTTCCCATGTG | 52 ^o^C | 30 |
| IMP (188bp) | FP: GGAATAGAGTGGCTTAAYTCTC  RP: CCAAACYACTASGTTATCT | 52 ^o^C | 30 |
| SPM (271bp) | FP: AAAATCTGGGTACGCAAACG  RP: ACATTATCCGCTGGAACAGG- | 52 ^o^C | 30 |
| *Class A carbapemases* | | 50 ^o^C | 30 |
| IMI/NMC-A (399bp) | FP: TGCGGTCGATTGGAGATAAA  RP: CGATTCTTGAAGCTTCTGCG |  | 30 |
| GES (323bp) | FP: GCGTGGTTAAGGATGAACAC  RP: CATCAAGTTCAACCCAACCG | 50 ^o^C | 30 |
| KPC(683bp) | PF: GTATCGCCGTCTAGTTCTGC  RP: 5'-GGTCGTGTTTCCCTTTAGCC | 52 ^o^C | 30 |
| SME (551bp) | FP: ACTTTGATGGGAGGATTGGC  RP: ACGAATTCGAGCATCACCAG | 52 ^o^C | 30 |
| *OXA carbapemases*  OXA (438bp) | FP: GCGTGGTTAAGGATGAACAC  RP: CATCAAGTTCAACCCAACCG | 63 ^o^C | 30 |
|  |  |  |  |
| *K. pneumoniae MLST* | |  |  |
| rpoB | FP: GGC GAA ATG GCW GAG AAC CA  RP: GAG TCT TCG AAG TTG TAA CC | 50 ^o^C | 30 |
| gapA | FP: TGA AAT ATG ACT CCA CTC ACG G | 60 ^o^C | 30 |
|  | RP:CTT CAG AAG CGG CTT TGA TGG CTT |  |  |
| *Mdh* | FP:CCC AAC TCG CTT CAG GTT CAG | 50 ^o^C | 30 |
|  | RP: CCG TTT TTC CCC AGC AGC AG |  |  |
| pgi | FP:GAG AAA AAC CTG CCT GTA CTG CTG GC | 50 ^o^C | 30 |
|  | RP:CGC GCC ACG CTT TAT AGC GGT TAA T |  |  |
|  | FP2:CTG CTG GCG CTG ATC GGC AT | Sequencing |  |
|  | RP2: TA TAG CGG TTA ATC AGG CCG T | sequencing |  |
| phoE | FP:ACC TAC CGC AAC ACC GAC TTC TTC GG | 50 ^o^C | 30 |
|  | RP:TGA TCA GAA CTG GTA GGT GAT |  |  |
| infB | FP:CTC GCT GCT GGA CTA TAT TCG | 50 ^o^C | 30 |
|  | RP:CGC TTT CAG CTC AAG AAC TTC |  |  |
|  | FP2:ACT AAG GTT GCC TCC GGC GAA GC | sequencing |  |
| tonB | FP:CTT TAT ACC TCG GTA CAT CAG GTT | 45^o^C | 30 |
|  | RP:ATT CGC CGG CTG RGC RGA GAG |  |  |
| *E.coli MLST* |  |  |  |
| *adk* | FP:ATTCTGCTTGGCGCTCCGGG | 95^o^C | 30 |
|  | RP:CCGTCAACTTTCGCGTATTT |  |  |
|  | FP2:TCATCATCTGCACTTTCCGC |  |  |
|  | RP2:CCAGATCAGCGCGAACTTCA |  |  |
| *FumC* | FP: TCACAGGTCGCCAGCGCTTC | 95^o^C | 30 |
|  | RP:TCCCGGCAGATAAGCTGTGG |  |  |
|  | RP2: GTACGCAGCGAAAAAGATTC | 95^o^C | 30 |
| *gyrB* | FP:TCGGCGACACGGATGACGGC |  |  |
|  | RP:GTCCATGTAGGCGTTCAGGG |  |  |
|  | FP:ATCAGGCCTTCACGCGCATC | 95^o^C | 30 |
| *Icd* | FP: ATGGAAAGTAAAGTAGTTGTTCCGGCACA |  |  |
|  | RP: GGACGCAGCAGGATCTGTT |  |  |
| *mdh* | FP:ATGAAAGTCGCAGTCCTCGGCGCTGCTGGCGG | 95^o^C | 30 |
|  | RP:TTAACGAACTCCTGCCCCAGAGCGATATCTTTCTT |  |  |
|  | FP2: AGCGCGTTCTGTTCAAATGC |  |  |
|  | RP: CAGGTTCAGAACTCTCTCTGT |  |  |
| *purA* | FP:TCGGTAACGGTGTTGTGCTG | 95^o^C | 30 |
|  | RP:CATACGGTAAGCCACGCAGA |  |  |
|  | FP2:TCGGTAACGGTGTTGTGCTG |  |  |
| *recA* | FP: CGCATTCGCTTTACCCTGACC | 95^o^C | 30 |
|  | RP:AGCGTGAAGGTAAAACCTGTG |  |  |
|  | FP2: AGCGTGAAGGTAAAACCTGTG |  |  |
|  |  |  |  |
